# Unexpected impact of radiation friction: enhancing production of longitudinal plasma waves

**DOI:** 10.1038/s41598-018-24930-x

**Published:** 2018-04-24

**Authors:** Evgeny Gelfer, Nina Elkina, Alexander Fedotov

**Affiliations:** 1 ELI Beamlines, Institute of Physics of the ASCR, v.v.i., Prague, Czech Republic; 2 National Research Nuclear University “MEPhI” (Moscow Engineering Physics Institute), Moscow, 115409 Russia; 3 Helmholtz-Institut Jena, Jena, 07743 Germany

## Abstract

We study the penetration of ultra-intense (intensity *I* $\simeq$ 10^23–24^ W/cm^2^) circularly polarized laser pulses into a thick subcritical plasma layer with accounting for radiation friction. We show that radiation pressure is enhanced due to radiation friction in the direction transverse to the laser pulse propagation, and that for stronger and longer laser pulses this mechanism dominates over the ordinary ponderomotive pressure, thus resulting in a substantionaly stronger charge separation than anticipated previously. We give estimates of the effect and compare them with the results of one and two dimensional particle-in-cell simulations. This effect can be important for laser-based acceleration schemes.

## Introduction

A new generation of 10 PW laser facilities (e.g., ELI Beamlines^1^, Apollon^2^, ELI NP^3^) will be soon commissioned around the world, providing very strong fields with intensity over 10^23^ W/cm^2^ and dimensionless amplitude ${a}_{0}=\frac{eE}{m\omega c}$ of the order of several hundreds. Here −*e* and *m* are electron charge and mass, *ω* is the laser carrier frequency, *E* is the electric field amplitude, and *c* is the speed of light. For ${a}_{0}\gg 1$ the electron quiver motion is already ultrarelativistic, but as *a*_0_ approaches few hundreds, it should become also strongly affected by radiation friction (RF)^4,5^.

For this reason, impacts of RF on various laser-plasma interaction processes and dynamics (nonlinear Thomson and Compton scattering^6–11^, inverse Faraday effect^12^, transformation of electron bunches crossing a laser pulse^13–16^, radiative trapping of electrons^17–19^, stimulated Raman scattering^20^, etc.) have received recently a substantial attention, see also the review^5^ and the recent experimental results^21–23^. Analytical solutions for a single particle motion with RF included are known^24–30^ for such simple cases as a constant magnetic field, a uniformly rotating electric field, and a plane wave field, however most of the research was performed using numerical simulations (different numerical approaches are compared in ref.^31^). One of the most promising applications of powerful lasers is ion acceleration in a plasma. The ions are accelerated by a quasistatic electric field arising because of the charge separation created by the laser pulse, for a review and recent experimental results see refs^32–34^.

In this paper we focus at the impact of RF on charge separation and longitudinal field generation^35^ by circularly polarized (CP) laser pulses propagating in a thick (the thickness is higher than the wavelength of the generated plasma wave) cold plasma with immobile ions. To facilitate penetration of electrons inside the high field region experiencing fully the action of RF force, we consider much lower plasma densities than in most of previous studies^36–40^. As a notable exception, it was shown^20^ that the stimulated Raman scattering in a low density plasma is substantially enhanced due to RF. It can still be shown that the characteristic time scale of the charge separation considered here is much shorter than the growth time of the instabilities considered in ref.^20^. As for generation of the longitudinal plasma waves, it was there out of the scope.

Longitudinal acceleration of an electron due to RF was already predicted in the literature^24–27^. However, here we apply it to charge separation in a plasma, and demonstrate both analytically and by numerical simulations, that RF can play a crucial role for propagation of a strong laser pulse in undercritical plasmas, resulting in substantial enhancement of both the amplitude and the period of a generated longitudinal wave.

## Results

**Figure 1:**
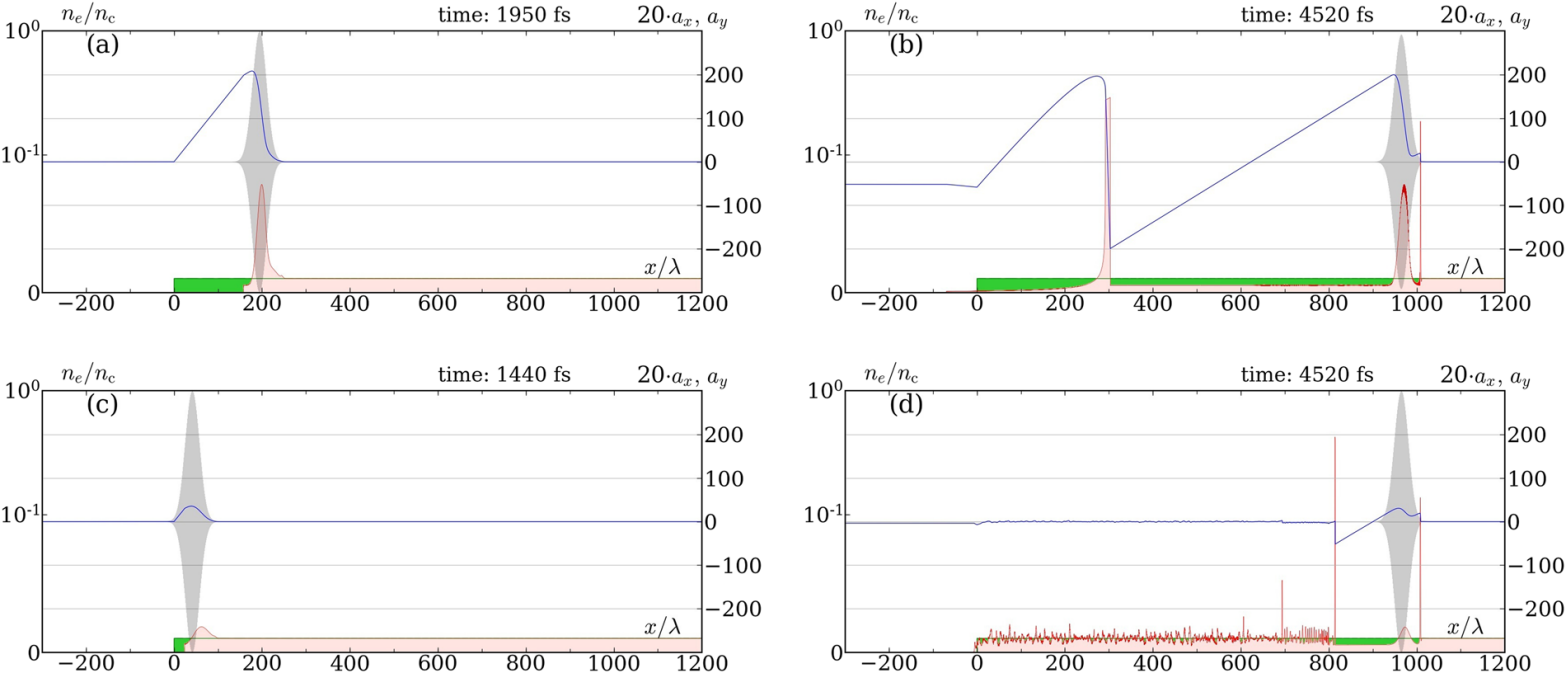
Successive snapshots of a circularly polarized laser pulse propagating through a 1D plasma with [(**a**,**b**)] and without [(**c**,**d**)] RF: red and green filled areas–electron and ion densities in units of critical density; grey line–*y* component of the dimensionless transverse electric field; blue curve–longitudinal component of the dimensionless electric field, multiplied by a factor of 20. Snapshots (**a**) and (**c**) are taken at the moments of first breakdown. Laser and plasma parameters are given in the text on p. 2.

To figure out the role of RF, let us start by presenting in Fig. 1 the results of 1D simulations of a laser pulse propagation in a cold plasma with and without RF. The parameters of a laser pulse are picked up according to the expectations of upcoming attainability, e.g., at ELI Beamlines^1^: peak envelope amplitude *a*_0_ = 300 (corresponding to the peak intensity *I*_L_ = 2.5 ⋅ 10^23^ W/cm^2^), full width half maximum (FWHM) 125 fs, and the wavelength *λ* = 1 *μ*m. The electron density of undisturbed plasma with ion charge number *Z* = 1 is *n* = 0.01*n*_*c*_, where *n*_*c*_ = *mω*^2^/4*πe*^2^ is the critical density.

As such a strong laser pulse enters the plasma, it grabs all the electrons on its front, no matter whether RF is taken into account or not. The resulting charge separation creates a quasistatic longitudinal electric field of strength ${E}_{\parallel }\,(x)=4\pi enx$ for 0 < *x* < *x*_m_, where *x*_m_ is the leftmost position of the shifted electrons. This field pulls back the electrons and its amplitude is growing as the pulse penetrates deeper into the plasma until a breakdown at *t* = *t*_bd_, when a bunch of electrons eventually penetrates back through the pulse, starting to accelerate against [see Fig. 1(a) and (c)].

Such bunches, generated at the successive breakdowns and running leftwards, partially screen the electrostatic field, thus bounding its amplitude, see Fig. 1(b) and (d). Comparing the two cases: when RF is turned on [Fig. 1(a,b)], and off [Fig. 1(c,d)], one observes that both the amplitude and the period of a created longitudinal plasma wave are much higher when RF is taken into account.

To explain the apparent paradox: radiation friction enhances longitudinal acceleration of the electrons^24–27^, we propose a model for the initial stage of the process before the first breakdown. Since $n\ll {n}_{c}$, let us consider motion of a single leftmost electron driven by the transverse field of the pulse

1 $${{\boldsymbol{a}}}_{\perp }={a}_{0}({\phi }\mathrm{)\{0,}\,\cos \,{\phi },\,\sin \,{\phi }\},\,{\phi }=\omega \,(t-x/c),$$

and the longitudinal field of the naked ions. To avoid possible confusion, we stress that by ***a*** we denote the dimensionless field strength rather than vector potential (if temporal envelope and RF are ignored, they are of the same magnitude, but directed perpendicular to each other).

In dimensionless variables, equation of electron longitudinal motion takes the form [see Methods for derivation from the Landau-Lifshitz Eq. (6)]:

2 $$\frac{d{u}_{x}}{d\tau }=\frac{1}{2\gamma }\frac{d{a}_{0}^{2}}{d{\phi }}+\mu {a}_{0}^{4}\frac{1-{\beta }_{x}}{1+{\beta }_{x}}-\xi \tilde{n},$$

where *τ* = *ωt*, *ξ* = *ωx*_m_/*c*, ***β*** = **v**/*c*, and *ñ* = *n*/*n*_*c*_ are the dimensionless time, position of the leftmost electron, electron velocity and density, respectively; *γ* is the electron Lorentz factor; **u** = *γ****β*** is the spatial component of the electron 4-velocity; $\mu =2\omega {r}_{e}\mathrm{/3}c\simeq 1.18\cdot {10}^{-8}$, and *r*_*e*_ = *e*^2^/*mc*^2^ is the classical electron radius. As explained in more details in Discussion, the first term on the RHS of (2) describes the ponderomotive force, while the second one originates due to modification of the electron transverse motion by RF. In what follows, we refer to them as the ponderomotive (PM) and the radiation friction (RFM) mechanisms of radiation pressure, respectively. Since radiation pressure results in a charge separation, one can also think of them as of competing mechanisms of charge separation.

Though Eq. (2) does not admit an exact solution, the process clearly splits into stages: (i) initially, as the pulse just starts penetrating into a plasma, the charge separation *ξ* is small, the electrostatic force is negligible, and the electrons are accelerated by the radiation pressure force; (ii) after some time *t*_acc_, when the electrostatic force counterbalances the radiation pressure force, the process enters the stage of steady deceleration and the LHS of Eq. (2) can be neglected; (iii) finally, a breakdown occurs, when a bunch of electrons finally penetrates to the rear of the pulse. Under an additional assumption that the longitudinal motion of electrons is ultrarelativistic, this splitting allows us to carry out a qualitative analysis and obtain analytical estimates (for technical details of their derivation see Methods).

Assuming that PM dominates over RFM, by neglecting the second term in the RHS of (2) corresponding to RFM, we arrive at the following estimates for the period and the amplitude of longitudinal wave generated solely by the ponderomotive mechanism (PM):

3 $${\tau }_{{\rm{bd}}}^{(\mathrm{PM})}\simeq {(\frac{{a}_{0}}{\tilde{n}\sqrt{T}})}^{\mathrm{2/3}},\,{a}_{\parallel }^{(\mathrm{PM})}\simeq \tilde{n}{\tau }_{{\rm{bd}}}^{(\mathrm{PM})}\simeq {a}_{0}^{\mathrm{2/3}}{(\frac{\tilde{n}}{T})}^{\mathrm{1/3}},$$

where *T* = *ωt*_p_ is the dimensionless pulse duration. Or else, assuming instead, that RFM dominates over PM, in the same way we arrive at

4 $${\tau }_{{\rm{bd}}}^{(\mathrm{RFM})}\simeq \sqrt{\frac{\mu T}{\tilde{n}}}{a}_{0}^{2},\,{a}_{\parallel }^{(\mathrm{RFM})}\simeq \sqrt{\mu \tilde{n}T}{a}_{0}^{2}\mathrm{.}$$

Equations (3) and (4) estimate the wavelength and the amplitude of the resulting longitudinal wave in the PM and RFM dominated regimes, respectively. They can be used, in particular, to conclude that RFM outperforms PM (${a}_{\parallel }^{(\mathrm{RFM})}\gtrsim {a}_{\parallel }^{(\mathrm{PM})}$) if

5 $${\mu }^{3}\tilde{n}{T}^{5}{a}_{0}^{8}\gtrsim \mathrm{1,}$$

i.e., for denser plasma and for stronger and longer pulses.

**Figure 2:**
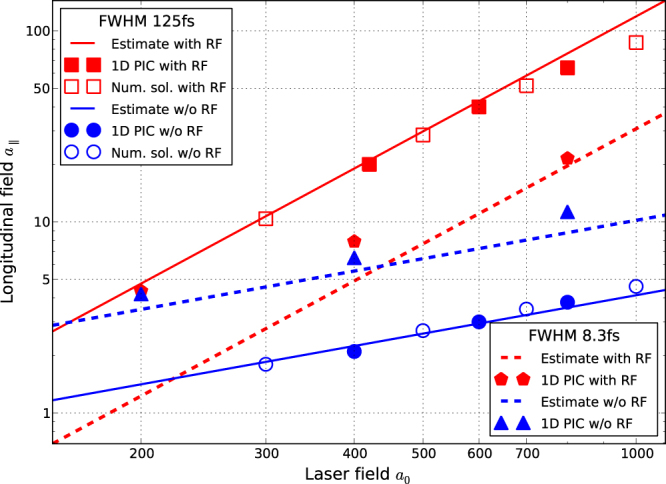
Amplitude ${a}_{\parallel }$ of a longitudinal wave generated due to RFM and PM by a CP laser pulse in a plasma with density *n* = 0.01*n*_*c*_ vs the amplitude *a*_0_ of the driving laser pulse, for two values of laser pulse duration (FWHM 125 fs and 8.3 fs).

In Fig. 2 we compare our estimates (3) and (4) (which with the adopted double-log scale appear as straight red and blue lines, respectively) to the numerical solution of the Landau-Lifshitz Eq. (6) (uncolored bullets), as well as to the results of 1D PIC simulation (filled bullets), each performed with and without RF–for short (FWHM = 8.3 fs) and long (FWHM = 125 fs) pulses. Corresponding data are shown in the same color. First of all, the figure shows that the corresponding markers for the numerical solution of our one-particle model (6) and for PIC simulations are distributed along the same lines. This was expected, as all the plasma effects but the charge separation should be negligible for the low densities considered thus far. Less trivially, as a rule the slopes of the lines and of the respective scatter data coincide, thus validating our estimates (3) and (4) up to numerical coefficients $\sim 1$. The only exceptions are the right upper square at the border of the applicability region (9), and the leftmost two pentagons shifted upwards from the dashed red line to the left of its crossing with the blue dashed line due to transition to the PM-dominated regime. Apart from that weak field region for a shorter pulse, and in the whole range of *a*_0_ for longer pulses, RFM clearly dominates over the PM. The established correspondence between the three approaches confirms both our estimates and the accuracy of our numerics.

**Figure 3:**
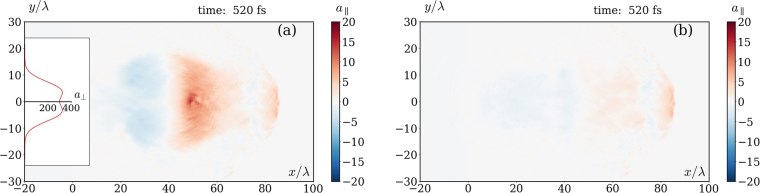
Longitudinal field distributions averaged over laser wavelength in 2D simulations of propagation of a CP laser pulse (with transverse profile along *x* = 0 shown in the inset) in a plasma with mobile ions ${}_{131}{}^{54}{\rm{X}}{\rm{e}}$^54+^: (**a**) with RF; (**b**) without RF [other parameters are given in the text on p. 5].

**Figure 4:**
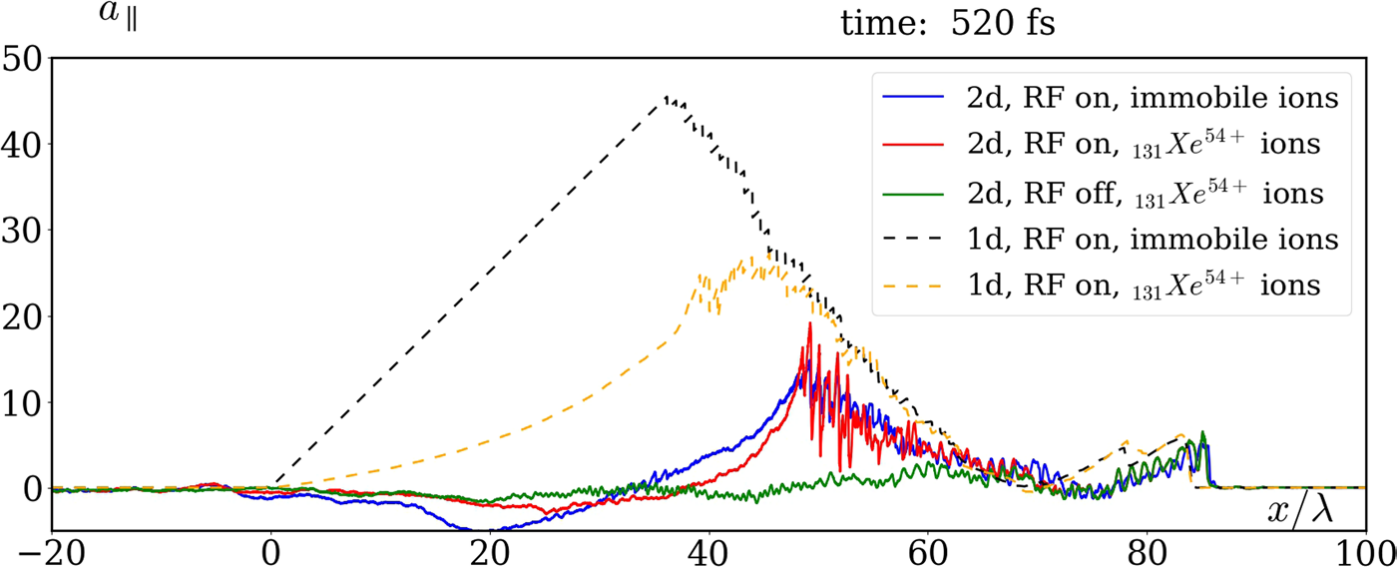
Comparison of longitudinal field on the *x*-axis in 2D simulations (solid lines) and in 1D simulations (dashed lines): with RF and immobile ions (blue and black), with RF and ${}_{131}{}^{54}{\rm{X}}{\rm{e}}$^54+^ ions (red and yellow), without RF and with ${}_{131}{}^{54}{\rm{X}}{\rm{e}}$^54+^ ions (green) [parameters are the same as in Fig. 3].

**Figure 5:**
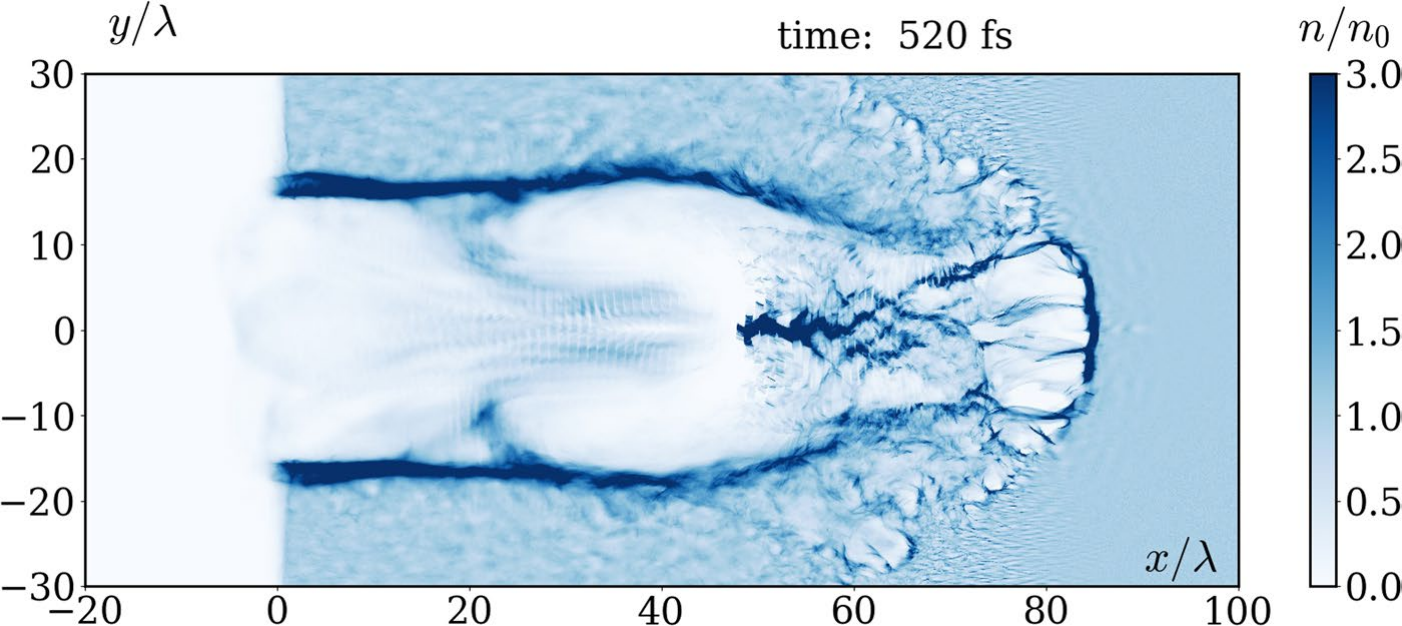
Electron density in 2D simulations of propagation of a CP laser pulse in a plasma with RF and mobile ions ${}_{131}{}^{54}{\rm{X}}{\rm{e}}$^54+^ [parameters are the same as in Fig. 3].

To substantiate the effect in a more realistic setup, we also made 2D EPOCH simulations with Xenon ions. Since ion acceleration results in a reduction of the charge separation and hence of the resulting longitudinal field, we assume the worst case that the ions are fully ionized, which according to the rough estimates based on ref.^41^ is very close to the actual expectations. The results for a symmetric bimodal Gaussian laser pulse [transverse profile is shown in the inset of the Fig. 3(a), see Methods for details] of intensity *a*_0_ = 300 (${I}_{L}\simeq 2.5\cdot {10}^{23}$ W/cm^2^), FWHM 125 fs and waist radius *w* = 5*λ* of each peak, focused at the left plasma boundary, are summarized in Figs 3, 4 and 5. We increased the plasma density *n* to 0.2*n*_*c*_ in order to strengthen the quasistatic longitudinal field on a background of the alternating longitudinal field of the pulse attributed to its tight focusing. The longitudinal fields computed with and without RF are compared in Fig. 3(a) and (b), where one can observe that the effect is extremely well pronounced in 2D. Moreover, the longitudinal field distribution on the *x*-axis is also in a qualitative agreement with 1D simulations, see Fig. 4. The most notable 2D effect is that a part of the electrons bypasses the ion bubble^42,43^, getting inside from its rear side [see Fig. 5], and in this way screening the quasistatic longitudinal field. Its decrease (as compared to the 1D simulation) at the rear of the resulting longitudinal wave in Fig. 4 is explained in part by this effect (compare the solid blue and the dashed black curves), and in the rest part by decrease of the charge separation gap due to ion motion (compare the red to blue and orange to black curves). Note that the difference between longitudinal fields obtained for mobile and immobile ions in 2D is substantially smaller than in 1D. The reason is that ions mobility in 2D is reduced due to partial screening of the field acting on them by the aforementioned electrons bypassing the bubble (compare blue to yellow curves).

## Discussion

One of the results of this paper is that there are in fact two different mechanisms of radiation pressure acting on electrons and, correspondingly, of charge separation in a laser plasma. This is manifested by Eq. (2) for electron longitudinal motion, where the first two terms on the RHS jointly describe the effect of radiation pressure [compare to Eq. (9) of ref.^40^]. The first one is the conventional relativistic ponderomotive force^44^, while the second one is induced by RF as follows: RF modifies the transverse quiver motion by additionally increasing the angle between the electron momentum and the magnetic field, thus enhancing the longitudinal component of the accelerating Lorentz force ∝ **v** × **B**^25^. The second term is precisely the resultant of this Lorentz force increment (directed forward) and the longitudinal RF force (directed backward). Alternatively, it can be understood as the net gain of momentum flux in a Thomson scattering occurring because the momenta of all the absorbed photons are parallel to *x* axis, while the scattered photons are bended by angles^45^ $\simeq$γ^−1^. The unusual stronger scaling $\propto \,{a}_{0}^{4}$ is due to the transverse electron motion^45,46^. To avoid possible confusion, let us stress that the RF-induced accelerating force [the second term on the RHS of Eq. (2)], though at first glance might seem reminiscent to the radiation pressure force $\propto \,{a}_{0}^{2}$ proposed^47^ to describe ion acceleration in the light sail regime, is in fact completely different, as is derivable from the Landau-Lifshitz equation for a dilute plasma without any account for plasma effects apart from the longitudinal charge separation field. In contrast, the radiation pressure considered in ref.^47^ originated as a combination of what we here call ponderomotive force, and the purely plasma effect of laser pulse reflection from the opaque plasma layer.

Our main results are the estimates (3) and (4) for the amplitude and the period of a longitudinal wave generated by laser pulses in plasma. They describe the regimes when this wave is produced by the ordinary ponderomotive mechanism, and by the radiation friction-induced mechanism just discussed, respectively. As shown in Fig. 2, they are in perfect agreement with the results of 1D PIC simulations with immobile ions in a wide range of parameters of the laser pulse. Since (4) contains a higher power of the laser field amplitude *a*_0_ than (3), and also since laser pulse duration *T* appears in numerator in the former and in denominator in the latter, we conclude that RFM outperforms PM for stronger and longer laser pulses, see Eq. (5) for a precise criterion. Plasma density also appears in (5), but in a lower power, hence the correspondent dependence is much weaker. According to PIC simulations, in 2D the enhancement of a quasistatic longitudinal field generation due to RFM is reduced due to electrons bypassing the ion bubble and, in addition, due to mobility of the ions (note that the latter effect in 2D is less substantial than in 1D). Although with this complications our estimates are no longer valid literally, they still remain qualitatively sound, and the effect still remains enough pronounced under these more realistic conditions.

To conclude, we propose a new RF-based mechanism of enhancement of the quasistatic longitudinal plasma field generation by laser pulses. Further development of our model by taking into account ion mobility and its application as a novel alternative mechanism for ion acceleration, will be given in a separate forthcoming publication.

## Methods

### Analytical Model

Consider the motion of the leftmost electron of the electron spike, see Fig. 1(a) and (c). Since plasma is dilute ($n\lesssim {n}_{c}$), this electron is driven by the three forces–(i) the Lorentz force from the laser transverse field (1), (ii) the longitudinal electrostatic force from the naked ions, and (iii) the RF force. Assuming also that both laser pulse and plasma are homogeneous in directions transverse to the laser pulse propagation, and that ions are immobile, according to Gauss’s law the dimensionless longitudinal electrostatic field equals to ${a}_{\parallel }=-\tilde{n}\xi$. Then, retaining only the dominant (∝ *γ*^2^) contribution to RF force in the Landau-Lifshitz form^45^, equations of motion in our dimensionless variables read

6 $$\begin{array}{l}\frac{d{{\bf{u}}}_{\perp }}{d\tau }=\mathrm{(1}-{\beta }_{x}){{\boldsymbol{a}}}_{\perp }-\mu {a}_{0}^{2}{\gamma }^{2}{\mathrm{(1}-{\beta }_{x})}^{2}{{\boldsymbol{\beta }}}_{\perp },\\ \frac{d{u}_{x}}{d\tau }={{\boldsymbol{a}}}_{\perp }{{\boldsymbol{\beta }}}_{\perp }-\mu {a}_{0}^{2}{\gamma }^{2}{\mathrm{(1}-{\beta }_{x})}^{2}{\beta }_{x}-\tilde{n}\xi \mathrm{.}\end{array}$$

Assuming that radiation damping is weak (${u}_{\perp }\approx {a}_{0}\gg 1$), we express the transverse field ***a***_⊥_ from the first of Eq. (6) and substitute it into the second one, and after a rearrangement using the relations *dφ* = (1 − *β*_*x*_)*dτ* and $\gamma ={\mathrm{(1}+{u}_{x}^{2}+{u}_{\perp }^{2})}^{\mathrm{1/2}}\equiv {\mathrm{[(1}+{u}_{\perp }^{2}\mathrm{)/(1}-{\beta }_{x}^{2})]}^{\mathrm{1/2}}$ we arrive at Eq. (2) for the longitudinal electron motion.

Equation (2) admits a substantial simplification if we further assume that longitudinal motion of the electron is ultrarelativistic [${u}_{x}\gg {u}_{\perp }\approx {a}_{0}$, *ξ*(*τ*) ≈ *τ*]. In this case, using *γ* ≈ *u*_*x*_, $1-{\beta }_{x}\approx {a}_{0}^{2}\mathrm{/2}{u}_{x}^{2}$, and 1 + *β*_*x*_ ≈ 2, we can reduce (2) to

7 $$\frac{d{u}_{x}}{d\tau }=\frac{1}{2{u}_{x}}\frac{d{a}_{0}^{2}}{d{\phi }}+\frac{\mu {a}_{0}^{6}}{4{u}_{x}^{2}}-\tau \tilde{n}\mathrm{.}$$

Besides, it turns out that in such a case the acceleration time ${t}_{{\rm{acc}}}\lesssim {t}_{{\rm{bd}}}$, hence the time of breakdown *t*_bd_ estimates the period of the resulting longitudinal plasma wave. The dimensionless time of breakdown *τ*_bd_ = *ωt*_bd_ is fixed by

8 $$T={\phi }({\tau }_{{\rm{bd}}})={\int }_{0}^{{\tau }_{{\rm{bd}}}}\mathrm{(1}-{\beta }_{x})d\tau \approx \frac{{a}_{0}^{2}}{2}{\int }_{0}^{{\tau }_{{\rm{bd}}}}\frac{d\tau }{{u}_{x}^{2}}\mathrm{.}$$

To obtain estimates (3) and (4), we assume in turn that one of the two competing mechanisms of radiation pressure (PM vs. RFM) dominates over the other. When PM is dominant, the second term on the RHS of Eq. (7) can be neglected. Then, with a suggestive estimate $d{a}_{0}^{2}/d{\phi }\sim {a}_{0}^{2}/T$ in Eq. (7), we have ${u}_{x}(\tau )\simeq {a}_{0}^{2}/T\tilde{n}\tau$ for the steady deceleration stage ${\tau }_{{\rm{acc}}}\lesssim \tau  < {\tau }_{{\rm{bd}}}$ and, substituting it further into Eq. (8), finally obtain (3). In the opposite case of RFM dominance, we drop the first term on the RHS in Eq. (7) and complete the rest of the estimates following the same lines. In particular, for the deceleration stage we have ${u}_{x}(\tau )\simeq {a}_{0}^{3}\sqrt{\mu /\tilde{n}\tau }$, and finally arrive at (4).

Let us also briefly comment on the restrictions validating the assumptions of our derivation. The first one (${u}_{\perp }\simeq {a}_{0}$), ensuring the weakness of the transverse motion damping due to RF, is that the transverse Lorentz force should substantially exceed the RF force. It turns out that this criterion can be formulated equivalently by that the energy stored in the resulting quasistatic longitudinal field remains much smaller than the total energy of the pulse, ${({a}_{\parallel }^{(\mathrm{RFM})})}^{2}{\tau }_{{\rm{bd}}}^{(\mathrm{RFM})}\ll {a}_{0}^{2}T$. The second restriction ${u}_{x}({\tau }_{{\rm{bd}}})\gtrsim {a}_{0}$ is needed to ensure that longitudinal electron motion is relativistic. Namely, for RFM, using Eq. (4), these restrictions can be formulated explicitly as

9 $${\mu }^{3}\tilde{n}T{a}_{0}^{8}\ll \mathrm{1,}\,\tilde{n}T/\mu {a}_{0}^{4}\lesssim 1.$$

For example, for $T\simeq {10}^{2}$ and 100 < *a*_0_ < 500 the restrictions (5) and (9) are fulfilled for $0.01\lesssim \tilde{n}\lesssim 1$, thus supporting our choice of the simulation parameters for this paper.

### Numerical Modelling

For numerical simulations we modified PIC code EPOCH^48^ by incorporating classical RF into its particle pusher. Namely, on each time step *i*, after calculating the particles momenta **p**(*i*) using the Boris algorithm^49^, they are additionally updated as^36,37^ ${\bf{p}}(i)\mapsto {\bf{p}}(i)+{{\bf{F}}}_{RR}(i-\mathrm{1)}{\rm{\Delta }}t$, where Δ*t* is the time step and **F**_*RR*_(*i* − 1) is the RF force, computed with the values from the previous step. We use only the highest^37^ second and third terms from the Landau and Lifshitz expression^45^ (this approach was also confirmed in ref.^31^). On our request, the preliminary 2D simulations (not presented in the paper) were also additionally verified with an alternative open source code SMILEI^50^.

The spatial resolution for all simulations was taken 100 cells/*λ*, and we used 20 and 10 particles per cell (with electrons:ions ratio 3:1) in 1D and 2D, respectively. Additional tests were performed to verify that the chosen resolution was sufficient to correctly determine the period and the amplitude of generating longitudinal wave. Note that apparent noise of a density distribution in Fig. 1 appears due to a two-stream instability. The instability arises after the electron bunches created on breakdowns reach the left plasma boundary, are turned around by the electrostatic field of the ions, and collide with the others.

In all simulations we consider a target as a thick cold plasma layer with a step left boundary. The temporal envelope of the laser pulses is Gaussian, exp(−*φ*^2^/*T* ^2^), with *T* = 283 (FWHM 125 fs) for long pulses in Figs 1–3 and *T* = 19 (FWHM 8.3 fs) for short pulses in Fig. 2. It turns out that in 2D an ordinary focused laser pulse in a low density plasma almost immediately expels electrons aside due to the transverse ponderomotive force. Because of that, the resulting longitudinal field is small and the difference between simulations with and without RF is almost negligible. Hence, in order to capture electrons inside a strong field region we used laser pulses with a symmetric bimodal Gaussian transverse spatial profile *G*(*x*, *y* − *d*/2) + *G*(*x*, *y* + *d*/2) shown in the inset of Fig. 3(a), where *G*(*x*, *y*) is an ordinary 2D Gaussian profile with waist radius *w* (see e.g. Eq. (2.30) of ref.^51^). The distance *d* ≈ 1.7*w* between the peaks is chosen so that the maximal field at the *x*-axis coincides to the peak envelope amplitude of each superposed pulses. For better visibility, the density distributions in Figs 1 and 5, as well as the field distributions in Fig. 3, were smeared out over laser wavelength.

### Data Availability Statement

The datasets generated and analyzed during the current study are available from the corresponding author on request.
